# Alzheimer’s disease increases the risk of erectile dysfunction independent of cardiovascular diseases: A mendelian randomization study

**DOI:** 10.1371/journal.pone.0303338

**Published:** 2024-06-13

**Authors:** Kaisen Liao, Qiang Lou

**Affiliations:** 1 Department of Urology, Tongde Hospital of Zhejiang Province, Hangzhou, Zhejiang, China; 2 Department of Andrology, the Second Affiliated Hospital of Guizhou University of Chinese Medicine, Guiyang, Guizhou, China; Tehran University of Medical Sciences, ISLAMIC REPUBLIC OF IRAN

## Abstract

**Background:**

Previous research has underscored the correlation between Alzheimer’s disease (AD) and erectile dysfunction (ED). However, due to inherent limitations of observational studies, the causative relationship remains inconclusive.

**Methods:**

Utilizing publicly available data from genome-wide association studies (GWAS) summary statistics, this study probed the potential causal association between AD and ED using univariate Mendelian randomization (MR). Further, the multivariable MR assessed the confounding effects of six cardiovascular diseases (CVDs). The primary approach employed was inverse variance weighted (IVW), supplemented by three additional methods. A series of sensitivity analyses were conducted to ensure the robustness of the results.

**Results:**

In the forward MR analysis, the IVW method revealed causal evidence of genetically predicted AD being a risk factor for ED (OR = 1.077, 95% CI 1.007∼1.152, *P* = 0.031). Reverse analysis did not demonstrate any causal evidence linking ED to AD (OR = 1.018, 95% CI 0.974∼1.063, *P* = 0.430). Multivariable MR analysis showed that after adjusting for coronary heart disease (OR = 1.082, 95% CI 0.009∼1.160, *P* = 0.027), myocardial infarction (OR = 1.085, 95% CI 1.012∼1.163, *P* = 0.022), atrial fibrillation (OR = 1.076, 95% CI 1.002∼1.154, *P* = 0.043), heart failure (OR = 1.103, 95% CI 1.024∼1.188, *P* = 0.010), ischemic stroke (OR = 1.079, 95% CI 1.009∼1.154, *P* = 0.027), hypertension (OR = 1.092, 95% CI 1.011∼1.180, *P* = 0.025), and all models (OR = 1.115, 95% CI 1.024∼1.214, *P* = 0.012), the causal association between AD and ED persisted. Sensitivity analyses confirmed the absence of pleiotropy, heterogeneity, and outliers, validating the robustness of our results (*P* > 0.05).

**Conclusions:**

This MR study consistently evidences a causal effect of genetically predicted AD on the risk of ED, independent of certain CVDs, yet offers no evidence for a reverse effect from ED.

## Introduction

Erectile dysfunction (ED) is defined as the inability to obtain or maintain a penile erection sufficient for achieving a satisfying sexual performance [1]. In the absence of any comorbidities, the prevalence of patients with ED increases from 10% to 79% from the age of 40 to 80, which affects millions of men around the world [2]. Epidemiologic studies show that ED is often accompanied by other pathologies such as diabetes, atherosclerosis, hyperlipidemia, metabolic syndrome, and Alzheimer’s disease (AD) [3]. Furthermore, the most common causes of ED are neurogenic and vascular factors, neurogenic factors promote ED at various levels of the nervous system from the local supply of the neuroautonomic system to the genital organ, and vascular factors contribute to ED mainly at the local supply. Briefly, the two factors interfere with the mechanisms that lead to cavernous smooth muscle relaxation, which is key to penile erection [4]. In addition, whether there are other factors that may affect ED remains to be further explored.

AD is a neurodegenerative condition that is genetically complex and associated with aging, characterized by progressive memory loss and cognitive decline. The pathogenesis of AD is relevance the formation of the neurotoxic oligomers of the amyloid-β peptide and neurofibrillary tangles in the brain that hamper proper neuronal functioning [5]. In AD, the most consistent association across all time points includes depression, ED, nervous and musculoskeletal symptoms [6]. The etiology and pathophysiology of AD and ED are thought to be associated with a neuropathic disease. Moreover, nitric oxide (NO)-guanylate cyclase (GC)-cyclic guanosine monophosphate (cGMP) signaling pathway play an important role in relaxation and penile erection. In detail, NO that released from nitrergic nerve endings and endothelial cells activates GC and increases cGMP, then the intracellular free Ca^2+^ decrease facilitating relaxation and penile erection [7]. Similarly, the activation of the NO related pathway ameliorates altered neuroplasticity and memory deficits in AD animal models [8]. Phosphodiesterase 5 (PDE5) inhibitors, including sildenafil and tadalafil, are widely used to treat conditions such as ED. Previous observational studies have found promising effects of PDE5 inhibitors in the treatment of myocardial infarction (MI), cardiac hypertrophy, heart failure (HF), cancer and anticancer drug-related cardiotoxicity, AD and other aging-related diseases [9, 10]. Although PDE5 inhibitors have been largely established in the treatment of ED, it is still unclear whether AD and cardiovascular factors are involved in the pathogenesis of ED.

In order to address methodological differences and elucidate the relationship of ED and AD, as well as possible cardiovascular disease (CVD) influencing factors, Mendelian randomization (MR) was used in our study. We evaluated the causal effect of genetic variants in European and East Asian cohorts by using single nucleotide polymorphisms (SNPs) as instrumental variables (IVs). Due to the random combination of SNPs during meiosis and resistance to external influences, the potential for conventional confounding variables is markedly reduced [11]. This method avoids the inherent defect of reverse causality in observational design. However, existing MR researches haven’t yet definitively established a causal link between ED and AD, leading to incomplete understanding. In this study, we investigate the causal association of AD and ED, underscoring the importance of establishing a clear causal pathway for timely interventions for patients with AD susceptible to ED.

## Materials and methods

The study included human participants who each signed a written informed consent form that underwent ethical review and approval in accordance with local legislation and institutional requirements. As the study utilized publicly available, anonymized, and de-identified genome-wide association study (GWAS) aggregated data, it was considered exempt from further ethical review approval.

### Study design

**Fig 1:**
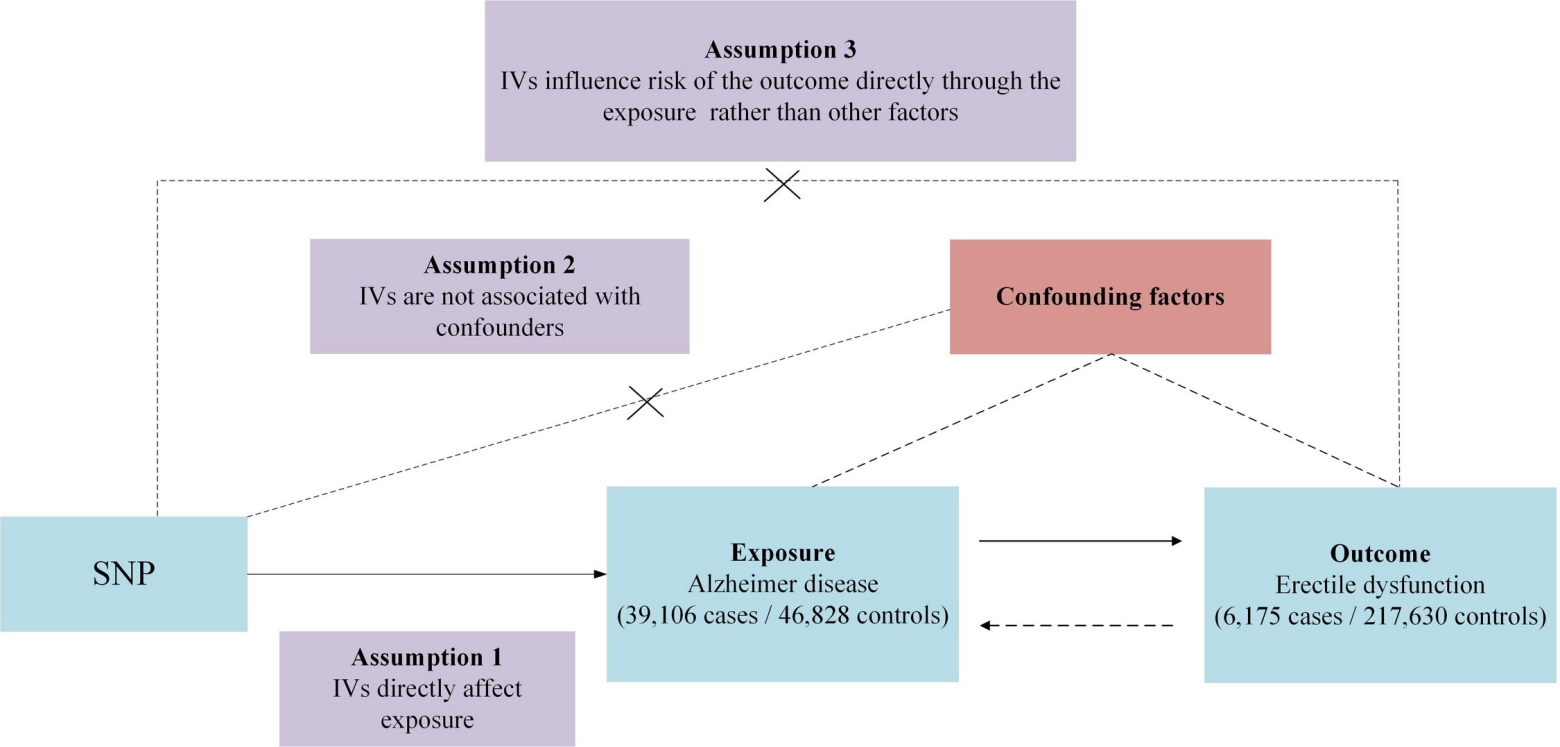
Overview of research design. The Mendelian randomization framework is based on three fundamental Mendelian randomization assumptions. IVs, instrumental variables; SNP, single nucleotide polymorphism.

This study relied on primary datasets obtained from publicly available, aggregate-level data from GWAS. Employing techniques such as univariate Mendelian randomization (UVMR) and multivariable Mendelian randomization (MVMR), we methodically evaluated the putative causal association between the exposure and the outcome. Selection of IVs for exposure adhered to three fundamental criteria: (i) the designated genetic marker, acting as the IV, demonstrates a robust association with the exposure; (ii) this particular genetic marker remains unlinked to potential confounding factors; and (iii) the influence of genetic variants on the outcome operates exclusively via the exposure, excluding other potential routes [12]. A detailed schematic of the MR methodology is illustrated in **Fig 1**. Comprehensive summary statistics derived from the data sources can be found in **Table 1**.

**Table 1:** Detailed information of data sources.

Phenotypes	Ref	Ieu id	Consortium	Ancestry	Participants
**Explore or Outcome**					
AD	35379992	ebi-a-GCST90027158	Bellenguez C	European	39,106 cases / 46,828 controls
ED	36402876	ebi-a-GCST006956	Bovijn J	European	6,175 cases / 217,630 controls
**Adjustment of the model**					
CHD	26343387	ieu-a-7	CARDIoGRAMplusC4D	77%European	60,801 cases / 123,504 controls
MI	26343387	ieu-a-798	CARDIoGRAMplusC4D	77%European	43,676 cases / 128,199 controls
AF	30061737	ebi-a-GCST006414	Nielsen JB	European	60,620 cases / 970,126 controls
HF	31919418	ebi-a-GCST009541	HERMES	European	47,309 cases / 930,014 controls
IS	34594039	ebi-a-GCST90018864	Sakaue S	European	11,929 cases / 472,192 controls
HTN	33959723	ebi-a-GCST90038604	Dönertaş HM	European	129,909 cases / 354,689 controls

Ref, reference (Pubmed id); HERMES, Heart failure Molecular Epidemiology for Therapeutic Targets; AD, Alzheimer disease; ED, Erectile dysfunction; CHD, Coronary heart disease; MI, Myocardial infarction; AF, Atrial fibrillation; HF, Heart failure; IS, Ischemic stroke; HTN, Hypertension; CARDIoGRAMplusC4D, Coronary Artery Disease Genome Wide Replication and Meta-analysis plus the Coronary Artery Disease Genetics.

### Selection of genetic instrumental variables

To ensure precise MR evaluations, we set forth rigorous criteria for our SNP selection: (i) For SNPs to qualify as IVs, it was imperative they demonstrated a genome-wide significant association with the pre-established exposure (*P* < 5×10^−8^). In the absence of genome-wide significant SNPs for ED phenotypes, we adopted a lenient threshold of 5×10–6, accommodating a broader range of SNPs for these phenotypes [13]. (ii) Selected SNPs underwent further refinement to negate potential associations with confounders and to guarantee their independence, minimizing biases from linkage disequilibrium (r^2^ < 0.001, clumping distance = 10,000 kb). (iii) We ascertained the legitimacy of our SNPs as IVs via F-statistics (F = beta²/se², where beta denotes the SNP-exposure association and variance is represented by se). Weak IVs were identified through this method [14]. An elevated F-statistic was indicative of robust instrumental validity, prompting us to stipulate an F-statistic exceeding 10 for all SNPs. (iv) To bolster the integrity of our findings, we employed MR-Steiger filtering, discarding variants more aligned with outcomes than with exposures [15]. (v) In instances where a SNP was missing from the outcome dataset, we engaged the SNiPa online platform (http://snipa.helmholtz-muenchen.de/snipa3/), anchored in European population genotype data sourced from the 1000 Genomes Project Phase 3. This facilitated the identification of the missing SNP and the subsequent location of a proxy SNP in linkage disequilibrium (criteria at r^2^ > 0.8) with the primary SNP. (vi) It was imperative that the SNP’s effect on both exposure and outcome consistently corresponded to an identical allele.

### Source of AD phenotype

The summary data for AD were sourced from the most recent and largest GWAS study to date, comprising 39,106 cases and 46,828 controls of European ancestry, contributed by the European Alzheimer & Dementia Biobank (EADB) consortium [16]. The EADB consortium represents a united initiative, amalgamating several European GWAS consortia focusing on AD. Meta-analysis of the EADB GWAS outcomes was conducted alongside the proxy-AD GWASs from the UK Biobank (UKBB) dataset. The UKBB defines its proxy-AD based on questionnaire responses where participants indicate if their parents suffered from dementia. The initial meta-analysis encompassed samples from these consortia/datasets: EADB, GR@ACE, EADI, GERAD/PERADES, DemGene, Bonn, Rotterdam, CCHS, NxC, and UKBB. Within the UKBB, those without any indication of dementia or a familial history thereof were designated as controls. The comprehensive analysis encompassed 2,447 clinically diagnosed cases, 46,828 proxy dementia cases, and 338,440 control subjects. All participants in the first stage were of European descent. The secondary stage incorporated samples from the Alzheimer’s Disease Genetics Consortium (ADGC), Cohorts for Heart and Aging Research in Genomic Epidemiology (CHARGE), and FinnGen consortia. Prior to participation, a formal written consent was secured from participants or, in instances of significant cognitive deficits, a responsible caregiver, legal guardian, or appointed representative. All cohort study protocols underwent scrutiny and received endorsement from the pertinent institutional review boards.

### Source of ED phenotype

The GWAS summary data for ED were derived from a meta-analysis conducted by Bovijn J et al., encompassing three cohorts and a total of 6,175 cases and 217,630 controls of European ancestry [17]. The diagnostic measures for ED were multifaceted, incorporating International Classification of Diseases, Tenth Edition (ICD-10) codes (N48.4 and F52.2), historical prescription records (including sildenafil), surgical intervention data, and self-reports from participants.

### Data sources for possible confounders

We further obtained genetic associations for atrial fibrillation (AF) from Nielsen JB et al [18]. Hypertension (HTN) associations were sourced from Dönertaş HM et al [19]. Ischemic stroke (IS) associations came from Sakaue S et al [20]. Data for HF were sourced from the Heart Failure Molecular Epidemiology for Therapeutic Targets (HERMES) [21], while coronary heart disease (CHD) and MI associations were obtained from the Coronary Artery Disease Genome-Wide Replication and Meta-analysis plus the Coronary Artery Disease Genetics (CardiogramplusC4D) [22].

### Statistical analyses

#### UVMR and MVMR analysis

Within the UVMR framework, the Wald ratio test was employed for individual IVs, while the multiplicative random-effects inverse variance weighted (IVW) method was integrated to ascertain causal associations across multiple IVs (≥2). This was supplemented by the MR-Egger and weighted median approaches. The IVW weighting corresponds with the Wald ratio estimate of each SNP and inversely correlates with its variance estimate [23]. Given the validity of all genetic variants, IVW produces consistent and efficient outcomes. In contrast, the weighted median approach is pertinent when over half of the genetic variants are deemed invalid, whereas MR-Egger presupposes total invalidity of such variants [24]. The constrained maximum likelihood (CML) method was also incorporated, facilitating simultaneous estimations across numerous genetic variants and accounting for potential confounders and genetic heterogeneity. CML delivers precise and resilient outcomes, particularly in the presence of extensive genetic variants and confounders [25]. In the MVMR analysis, to account for potential confounders in the exposure-outcome trajectory, MVMR investigations were conducted to elucidate the direct causal linkage between exposure and outcome. Unlike the UVMR model, the foundational tenet of MVMR posits that genetic variation aligns with one or multiple exposures, with subsequent postulations in line with UVMR conventions [26].

### Sensitivity analysis

In the UVMR framework, a rigorous set of methodological evaluations was undertaken. Heterogeneity across selected genetic variants was assessed using Cochran’s Q test, with a *P* -value less than 0.05 indicating notable variance among the scrutinized SNPs [27]. The presence of directional pleiotropy in the MR model was explored via MR-Egger regression [28]. An MR-Egger intercept *P* -value less than 0.05 denotes significant directional pleiotropy, given the intrinsic limitations of the technique [29]. The MR-Pleiotropy RESidual Sum and Outlier (MR-PRESSO) approach pinpointed outliers and evaluated horizontal pleiotropy, which is affirmed if the global *P* -value is less than 0.05 [30]. Outliers were diligently removed to refine our adjustments, and a subsequent leave-one-out analysis assessed the contribution of individual SNPs to the aggregate results [31].

R^2^ was derived from 2×MAF×(1-MAF)×beta^2^, wherein MAF denotes the minor allele frequency of each incorporated SNP. These derived values were amalgamated to yield the requisite coefficient for power estimation [32]. The statistical power was ascertained via utilities from the mRnd website [33] (https://shiny.cnsgenomics.com/mRnd/).Additionally, we employed online tools to assess the bias introduced by sample overlap (https://sb452.shinyapps.io/overlap/), and the results yielded a “Bias”of less than 0.001, confirming the robustness of our findings [34].

## Results

### Genetic instrument selection

Within the GWAS data for AD and ED, the number of SNPs selected as IVs for AD and ED were 57 **(S1 Table)** and 10 **(S2 Table)**, respectively. The study demonstrated that the F-statistics for all IVs exceed 25, indicating a substantial mitigation of bias due to weak IVs. The variance explained was 17.95% for AD and 7.36% for ED **(S3 Table)**. All IVs passed the MR-Steiger filtering, and the direction of analysis was consistent, adhering to the third assumption of MR.

### Association of genetically predicted AD with ED

**Fig 2:**
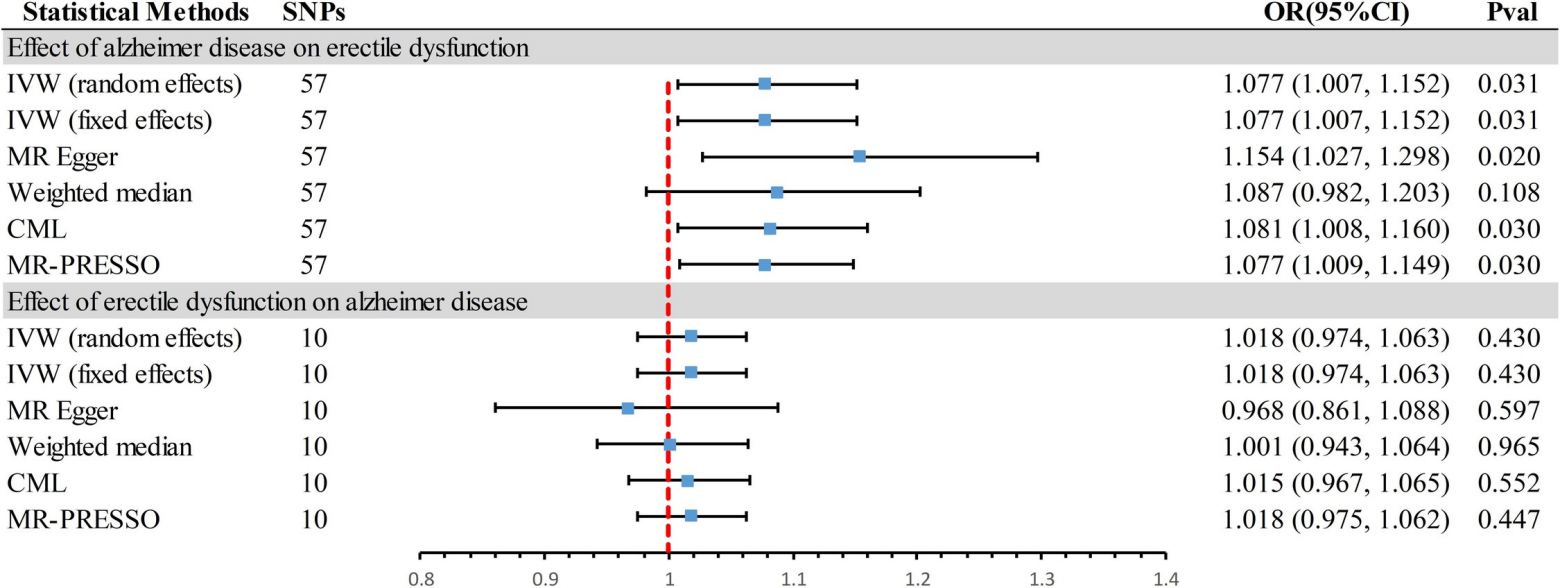
Forest plots showed the causal associations between Alzheimer’s disease and erectile dysfunction. IVW, inverse variance weighted; CI, confidence interval; OR, odds ratio; SNPs, single nucleotide polymorphisms; CML, constrained maximum likelihood; MR- PRESSO, Mendelian randomization-Pleiotropy RESidual Sum and Outlier.

**Fig 3:**
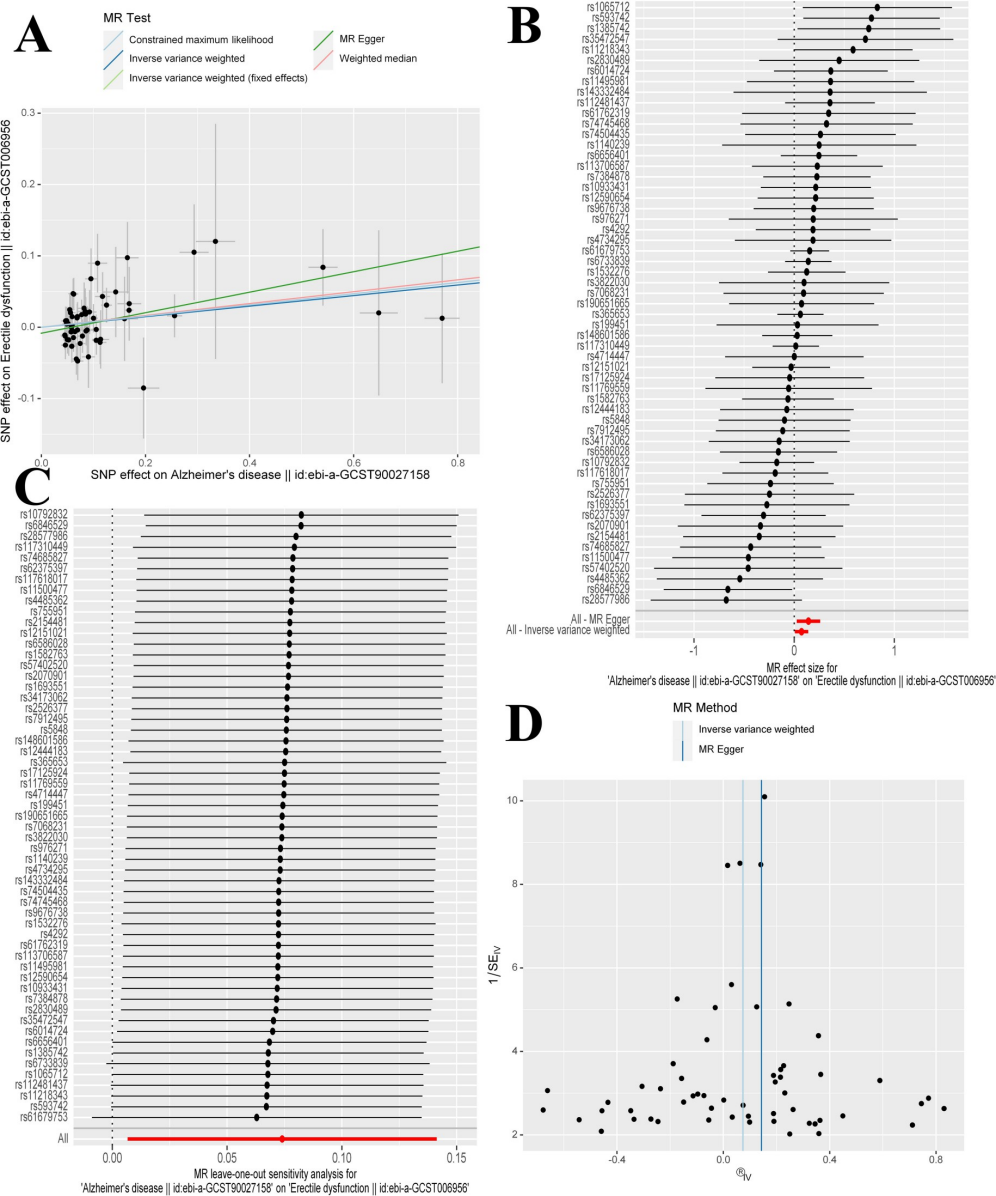
The causality of Alzheimer disease on erectile dysfunction. (A) Scatter plot (B) Forest plot (C)Leave-one-out (D) Funnel plot. MR, Mendelian randomization; SNP, single nucleotide polymorphism.

In the forward MR analysis **(Fig 2)**, significant causal evidence was found suggesting that AD is a risk factor for ED. Specifically, the primary IVW method indicated that for every standard deviation (SD) unit increase in genetically predicted AD, the incidence of ED increased by 7.7%. This finding was consistent between random and fixed effects [odds ratio (OR) = 1.077, 95% confidence interval (CI) 1.007∼1.152, *P* = 0.031] models. Supplementary methods, including MR Egger (OR = 1.154, 95% CI 1.027∼1.298, *P* = 0.020) and CML (OR = 1.081, 95% CI 1.008∼1.160, *P* = 0.030), provided corroborative evidence for this association. With an OR of 1.077, we achieved a statistical power of 75% to detect an association between AD and ED. Scatter plots provide a visual representation of all the results **(Fig 3A)**, while forest plots detail the causal effect offered by each IV **(Fig 3B)**. In sensitivity analyses **(Table 2)**, MR-PRESSO detected no outliers and presented no evidence of potential directional pleiotropy (OR = 1.077, 95% CI 1.009∼1.149, *P* = 0.030, P_global test_ = 0.630). No heterogeneity was identified by the Cochran’s Q statistic (*P* > 0.05), and MR-Egger showed no evidence of pleiotropy (*P* > 0.05). The leave-one-out analysis confirmed that the causal association was not driven by a single SNP **(Fig 3C)**, and funnel plots demonstrated symmetry **(Fig 3D)**.

**Table 2:** Summary of sensitivity results.

Exposure	Outcome	MR-Egger intercept			MR-PRESSO global test			Cochrane’s Q			Steiger_test	
		Intercept	SE	*Pval*	RSS_obs_	*P*-value	*Outlier*	*Q*	*Q_df*	*Q_pval*	Direction	*Pval*
AD	ED	-0.008	0.006	0.161	53.730	*0*.*630*	*NA*	52.270	56	0.617	TRUE	0
ED	AD	0.010	0.011	0.391	13.344	*0*.*342*	*NA*	8.852	9	0.451	TRUE	4.67E-09

MR, Mendelian Randomization; MR-PRESSO, Mendelian randomization-Pleiotropy RESidual Sum and Outlier; AD, Alzheimer disease; ED, Erectile dysfunction.

In the reverse MR analysis **(Fig 2)**, both the primary methods of IVW random and fixed-effects (OR = 1.018, 95% CI 0.974∼1.063, *P* = 0.430) models suggested no evidence of a causal association between ED and AD, refuting the reverse causality hypothesis. Supplementary methods, including MR Egger, Weighted median, and CML, yielded consistent results with the primary methods. In the reverse analysis, the limited sample size precluded achieving sufficient statistical power sensitivity analyses revealed no evidence of pleiotropy or heterogeneity **(Table 2)**. All figures can be found in **S1 Fig**.

**Fig 4:**
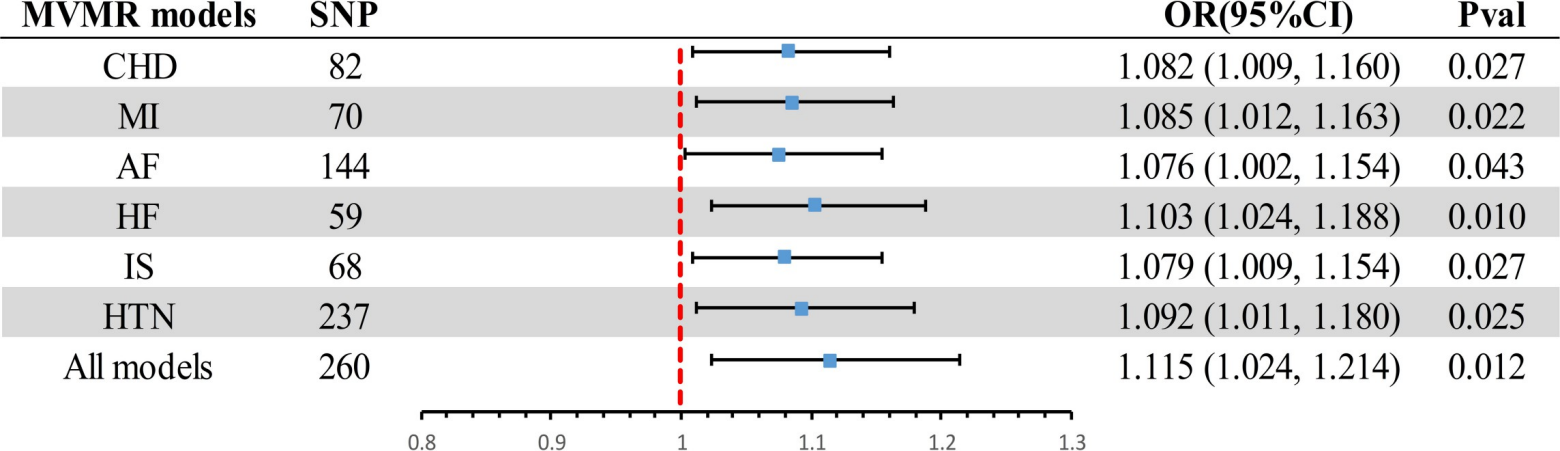
MVMR analyses the causal effect of potential confounding cardiovascular disease in Alzheimer’s disease on erectile dysfunction. MVMR, Multivariable Mendelian randomization; CI, confidence interval; OR, odds ratio; SNP, single nucleotide polymorphism; CHD, coronary heart disease; AF, atrial fibrillation; HTN, hypertension; IS, ischemic stroke; HF, heart failure MI, myocardial infarction.

In the UVMR analysis, the findings provide evidence supporting a causal association between AD and ED as a risk factor, passing the statistical significance threshold (*P* < 0.05). In the MVMR analysis **(Fig 4 and S4 Table)**, after accounting for potential confounding CVDs: CHD (OR = 1.082, 95% CI 0.009∼1.160, *P* = 0.027), MI (OR = 1.085, 95% CI 1.012∼1.163, *P* = 0.022), AF (OR = 1.076, 95% CI 1.002∼1.154, *P* = 0.043), HF (OR = 1.103, 95% CI 1.024∼1.188, *P* = 0.010), IS (OR = 1.079, 95% CI 1.009∼1.154, *P* = 0.027), and HTN (OR = 1.092, 95% CI 1.011∼1.180, *P* = 0.025), and adjusting all models (OR = 1.115, 95% CI 1.024∼1.214, *P* = 0.012), the association remains significant. The MVMR results further demonstrated that AD is a risk factor for ED, independent of CVDs.

## Discussion

This MR study comprehensively validated the causal association between AD and ED using multiple models and identified AD as a risk factor for ED. Further MVMR suggests that the risk causal effect of AD on ED is independent of the six CVDs. Reverse MR analysis showed that ED had no causal relationship with AD. The penile erection process contains three regions including the central nervous system, the autonomous nervous system and genital organs. We will compare the effects of AD on penile erection in these regions.

Our findings of AD were consistent with previous researches. Compared to psychological factors (anxiety, stress, and mental disorders) and increasing age, neurological and vascular are the main factors leading to ED [35]. Neurological regulation of erectile function involves a complex network of primary afferents, spinal interneurons, sympathetic nerves, and parasympathetic nerves. Neurological disorders such as AD may result in endocrine system or cardiovascular system abnormalities, which can have an impact on sexual function. Briefly, the pathogenesis of AD includes neuroinflammatory plaques formed by the deposition of β-amyloid plaques outside nerve cells and neurofibrillary tangles formed by aggregation of hyperphosphorylated tau protein inside nerve cells, resulting in neuroinflammation, neuronal and synaptic loss [36, 37]. Moreover, the neuropathy caused by AD was involved in the penile erection process. In detail, the penile erection process contains the following steps. First, the central nervous system receives sexual stimulation, such as visual, auditory, olfactory and tactile. Then the sexual information transfer to the spinal cord and the autonomous nervous system through the activated signal pathways. Finally, the genital organs receive the signal to induce penile erection [1].

The hypothalamus plays a key role in the central nervous system of penile erection [38], which can triggers the erectile response evoked by erotic clips [39]. For instance, Cera et al. found the presence of macro structural changes in grey matter of the nucleus accumbens and the hypothalamus in ED patients with respect to controls. Meanwhile, a grey matter atrophy of left hypothalamus was correlated with low erectile function tested by international index of erectile function [40]. Moreover, the hypothalamic-pituitary axis could regulate the hormonal production that could obtain or maintain erectile function. Sustained high levels of prolactin lead to the development of male sexual dysfunction and the regulation of prolactin release occurs primarily in the hypothalamus, whose dopaminergic neurons inhibit the activity of lactotroph cells [41]. Similarly, oxytocin that has important role in mental protective adaptations and sexual emotions lending the name of love hormone is synthesized in the hypothalamus and secreted by the posterior pituitary [42]. However, AD patients showed a neurofibrillary tangles in the hypothalamus and exhibit abnormal changes in the hypothalamic-pituitary-adrenal axis. Compared with healthy controls, AD patients with depression showed reduced functional connectivity among the hypothalamus by using magnetic resonance imaging method [43]. As the AD disease worsens, β-amyloid plaques and neurofibrillary tangles were deposited in several hypothalamic nuclei, such as the suprachiamastic nucleus, the supraoptic nucleus and the paraventricular nucleus [44, 45]. And reduced hypothalamic volume and gray matter loss was also observed [46]. Some post-mortem human studies showed that aberrant protein spreading and neuroinflammation may cause hypothalamus degeneration [47]. Zhang et al. discovered that the abnormal metabolism in the hypothalamus may serve as a potential biomarker for the early detection and monitoring of amyloid pathology progression in mouse models [45]. All the AD-related studies suggested that the hypothalamus is damaged in AD patients, affecting its normal endocrine and autonomic function including the key role in the central nervous system of penile erection.

As an extension of the brain, the spinal cord acts as a bridge between the brain and genital organs, which requires an entire neural pathway like autonomic nervous system. More importantly, any lesion to the neural pathway that interrupts the transmission of signals may potentially induce ED [48]. However, the bridge might be rendered useless by the β-amyloid deposition and hyperphosphorylated tau in the spinal cord occurs during the preclinical stage of AD. For example, Yuan et al. reported β-amyloid deposition which distribution was corresponds to the corticospinal tract pathway, occurs in the spinal cord of AD mice [49]. Dugger et al. investigated that phosphorylated tau was detected in the cervical cord segments 96% of 46 patients with AD. In detail, a lower percentage of phosphorylated tau was also detected in the thoracic spinal segment, lumbar spinal segment, and sacral spinal segment [50]. Moreover, Hyperphosphorylated tau protein was found in spinal anterior horn cells, glial cells, and axons [51], and neurofibrillary tangles caused by hyperphosphorylated tau protein was observed in each segment of the spinal cord in patients with AD [52], indicating that the pathological changes involving the autonomic nervous system could occur in the spinal cord of patients with AD. Thus the diseased spinal cord and autonomous nervous system might not be able to complete the transmission of sexual information from the brain.

When sexual stimulation is transmitted through the nerves to the genital organs, cholinergic inhibitory and nonadrenergic noncholinergic inhibitory causes relaxation of cavernous smooth muscles and facilitates erection. In NO-GC-cGMP signaling pathway, NO released from nitrergic nerve endings and from endothelial cells containing endothelial NO synthase. Then cGMP acted on protein kinase GK1 that could decrease the concentration of intracellular Ca^2+^ ions. In AD, endothelial and inducible nitric oxide synthase were highly expressed in astrocytes, leading to high concentrations of NO, which exhibited toxicity and contributed to peroxynitrite formation, resulting in protein nitration and nitrosylation, lipid and DNA damage, and neuronal cell death [53]. Furthermore, a decrease of soluble GC expression in reactive astrocytes surrounding the β-amyloid deposition in AD brains was observed, which might be a mechanism to prevent excess signaling via cGMP at sites of high NO production [54], suggesting NO-GC-cGMP signaling pathway was disturbed and the relaxation of cavernous smooth muscles was impeded in AD [55].

AD is still not completely cured, but it is possible to prevent and delay disease progression. Previous studies have highlighted the link between AD and CVD [56–58]. Cardiovascular risk factors are prevalent in older adults, and there are conflicting reports about the association between modifiable cardiovascular risk factors and AD. The mechanism of these associations is uncertain, but may be the result of a direct combination with mechanisms related to cerebrovascular disease. Research has also shown that high blood pressure, hyperlipidemia, hyperhomocysteinemia and smoking are potentially important risk factors for AD [59]. Coronary artery bypass grafting and transplant surgery, also appear to increase the risk of AD. These diseases share some common biological pathways, and apolipoprotein E is a major risk factor for AD. Apolipoprotein E does not appear to cause AD by increasing serum cholesterol, but it may contribute to the disease through mechanisms involving Abeta and increased vulnerability of neurons to stress. Aggressive treatment of CVD may be useful in treating or preventing AD. Statins may help prevent the progression of dementia in AD patients [56]. There are also studies emphasizing the association between CVD and ED [60–62]. ED is a very common condition, especially in men with cardiovascular damage. Many of the recognized risk factors for CVD are also risk factors for ED. The correlation between ED and endothelial dysfunction has been fully confirmed, and due to impaired smooth muscle endothelial dependent diastolic function, there is an association between ED and ischemic heart disease.[63]. Increasing evidence suggests that ED may be a sentinel event for CVD [61, 63]. ED may be a clinical marker of vascular disease and a predictor of the progression of CVD [60].

Given the important role of CVDs in the relationship between AD and ED, we preliminarily estimated that CVDs could be confounding factors in this association and performed MVMR analysis. The results show that although AD is a risk factor for ED, this causality is not affected by the six CVDs, including CHD, MI, AF, HF, IS, and HTN. The research on the relationship between CVD and AD and ED is mostly based on case reports or clinical studies, which may lead to differential results. The causal sequence between CVD and their onset is not clear, and may also be one of the reasons for differences. In the future, more research can be conducted on the relationship between CVD and AD and ED, whether from clinical and basic research, or research on the causal relationship of disease mechanisms.

Our research has several advantages. Firstly, multiple models were used to verify the causal effect in multiple dimensions to minimize the bias of the results. Second, all F-values of SNP were greater than 10, avoiding weak tool bias. Third, this is the first MR analysis to elucidate the causality of AD and ED. Fourth, MVMR confirms that AD acts on ED independently of the six CVDs. Fifth, in our sensitivity analysis, we used different statistical models to assess the robustness of our primary findings and improve the reliability of the evidence.

Admittedly, there are some limitations of our research. First, the research was conducted in a European population, so it is uncertain whether the findings apply to other populations. Therefore, future researches on the causal relationship between AD and ED should include population samples from different ethnicities to improve the generalizability of the results. Second, the aggregated data cannot be analyzed in hierarchical subgroups. As a result, future studies could explore these subpopulations in more detail.

## Conclusion

This study is the first to explore the genetic causal relationship between AD and ED. The findings suggest that AD can increase the risk of ED and is independent of certain CVDs. However, there is no evidence that ED has an effect on AD. These findings can provide valuable insights into the relationship between AD and ED at the genetic level.

## Supporting information

S1 Table Instrumental variables for Mendelian randomization analysis of all Alzheimer’s diseases. (XLSX)

S2 Table Instrumental variables for Mendelian randomization analysis of all erectile dysfunctions. (XLSX)

S3 Table Power calculations for bidirectional univariable Mendelian randomization analyses. (XLSX)

S4 Table Summary of analytical results for MVMR. (XLSX)

S1 Fig The causality of erectile dysfunction on Alzheimer disease. (A) Scatter plot (B) Funnel plot (C)Leave-one-out (D) Forest plot. MR, Mendelian randomization; SNP, single nucleotide polymorphism.
(JPG)
