# Supplementary figures and images for: Alzheimer’s disease increases the risk of erectile dysfunction independent of cardiovascular diseases: A mendelian randomization study
Source: PLoS One. 2024 Jun 13;19(6):e0303338. doi: 10.1371/journal.pone.0303338 (PMC11175418; doi:10.1371/journal.pone.0303338)

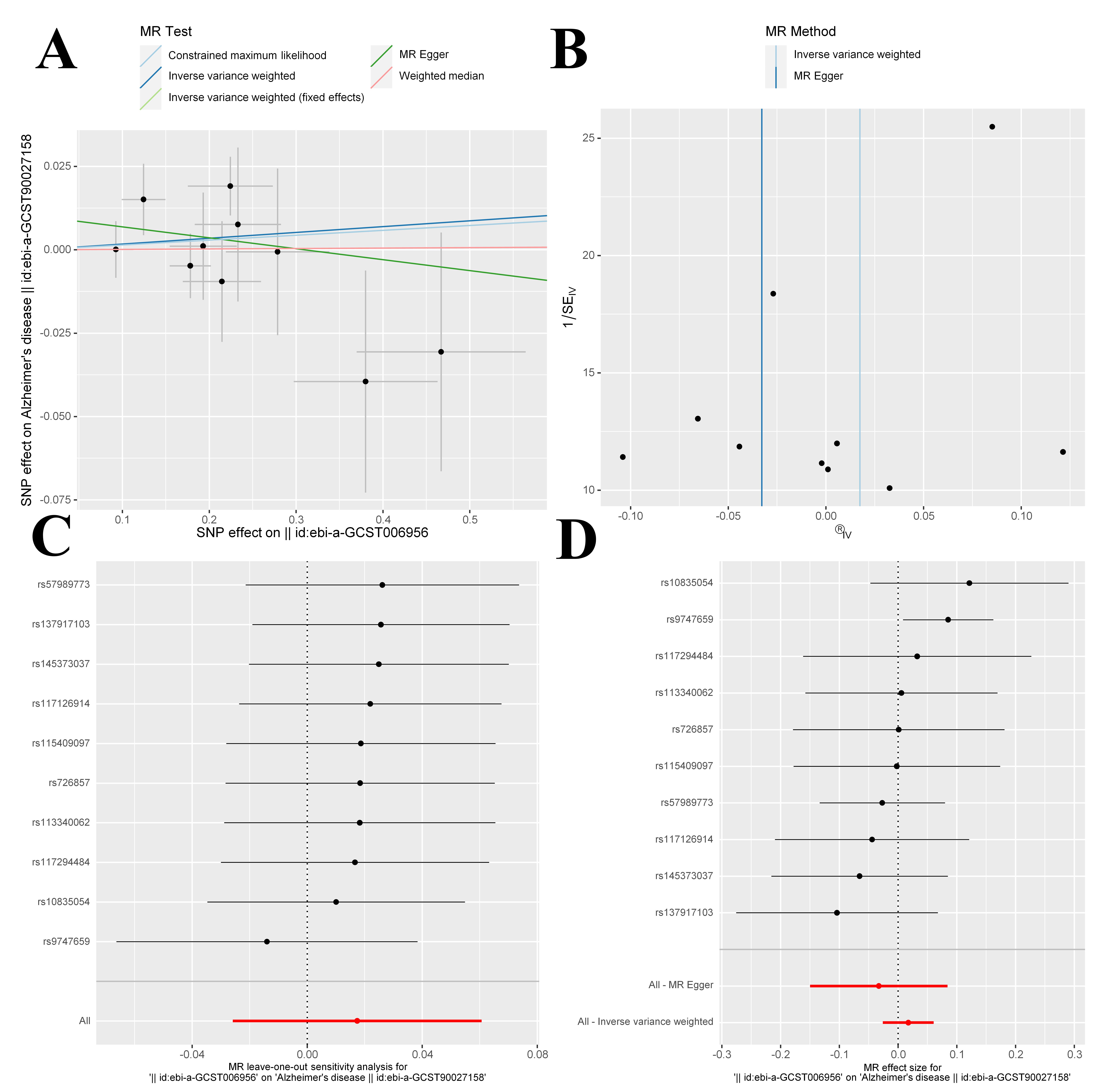

Supplement: S1 Fig — (A) Scatter plot (B) Funnel plot (C)Leave-one-out (D) Forest plot. MR, Mendelian randomization; SNP, single nucleotide polymorphism. (JPG) [file pone.0303338.s005.jpg]
